# 가상현실을 이용한 모아간호 실습교육이 간호 대학생의 실습역량에 미치는 영향: 체계적 문헌고찰

**DOI:** 10.4069/kjwhn.2022.09.13

**Published:** 2022-09-30

**Authors:** Sungwoo Hwang, Hyun Kyoung Kim

**Affiliations:** 1 Department of Nursing, Baekseok Culture University, Cheonan, Korea; 1 백석문화대학교 간호학과; 2 Department of Nursing, Kongju National University, 56 Gongjudaehak-ro, Gongju, Korea; 2 공주대학교 간호학과

**Keywords:** Maternal-child nursing, Nursing education, Nursing students, Systematic review, Virtual reality, 모아간호, 간호교육, 간호학생, 체계적 문헌고찰, 가상현실

## Abstract

**Purpose:**

This study aimed to investigate the effects of virtual reality used in maternal-child nursing clinical practicums on nursing students’ competencies through a systematic review.

**Methods:**

The inclusion criteria were peer-reviewed papers in English or Korean presenting analytic studies of maternal-child nursing practicums using virtual reality. An electronic literature search of the Cochrane Library, CINAHL, EMBASE, ERIC, PubMed, and Research Information Sharing System databases was performed using combinations of the keywords “nursing student,” “virtual reality,” “augmented reality,” “mixed reality,” and “virtual simulation” from February 4 to 15, 2022. Quality appraisal was performed using the RoB 2 and ROBINS-I tools for randomized controlled trials (RCTs) and non-RCTs, respectively.

**Results:**

Of the seven articles identified, the RCT study (n=1) was deemed to have a high risk of bias, with some items indeterminable due to a lack of reported details. Most of the non-RCT studies (n=6) had a moderate or serious risk of bias related to selection and measurement issues. Clinical education using virtual reality had positive effects on knowledge, skills, satisfaction, self-efficacy, and needs improvement; however, it did not affect critical thinking or self-directed learning.

**Conclusion:**

This study demonstrated that using virtual reality for maternal-child nursing clinical practicums had educational effects on a variety of students’ competencies. Considering the challenges of providing direct care in clinical practicums, virtual reality can be a viable tool that supplements maternal-child nursing experience. Greater rigor and fuller reporting of study details are required for future research.

## Introduction

4차 산업혁명의 물결이 교육 분야에도 빠르게 확산하여 다양한 정보통신 기반 프로그램이 적용되고 있다. 특히, 가상현실(virtual reality, VR)을 이용한 교육은 직접 체험하기에는 위험하거나 비용이 많이 드는 교육 및 훈련을 현실과 유사하게 설계된 가상의 공간에서 간접적으로 체험함으로써 다양한 교육적 경험을 얻을 수 있도록 하여 시간적, 공간적 제약에서 벗어나도록 하고 있다[1]. 향후 실감 콘텐츠 기술의 지속적 발전은 미래의 교육환경을 혁신적으로 변화시킬 것으로 전망된다[2].

간호교육 분야는 이론을 기반으로 임상현장에서 대상자의 상황에 맞게 지식을 총체적으로 적용하고 문제를 해결할 수 있는 역량을 키우는 현장 실습교육이 매우 중요하다[3]. 그러나 최근 COVID-19 감염병으로 인해 대면 현장 실습교육이 불가한 경우가 발생하고 있으며, 교내 실습교육 역시 제한적으로 운영되고 있는 실정이다[4]. 저출산 사회현상으로 인한 분만 건수 급감으로 우수한 모아간호(maternal-child) 실습 기관 확보가 매우 어려운 상황이며[5], 환자의 인권 보호와 사생활 존중 문화 확산으로 임상에서는 관찰 위주의 실습이 운영되고 있다[6]. 현 상황은 실습교육의 제한점을 해결하기 위해 다양한 대체 실습프로그램이 고안되고 있고[7-9], 가상현실을 이용한 실습교육 중재를 시도하고 있는 시점이므로 실습역량에 미치는 효과를 고찰할 필요가 있다[10].

간호역량(nursing competency)은 전문적 판단, 기술, 가치와 태도를 포함한 지식의 통합 능력으로 정의할 수 있다[11]. 간호 대학생의 실습역량은 지식, 기술, 태도의 속성으로 분류하고 측정할 수 있다[12]. 가상현실을 활용한 실습교육이 간호 대학생의 실습역량에 미치는 선행연구를 살펴보면, 헤드셋을 활용한 술기교육은 실습 후 간호 대학생의 학습 자기효능감을 향상시켰고[13], 360도 카메라로 병원환경을 촬영한 영상의 활용은 간호 대학생의 지식, 교육만족도, 학업성취도를 향상시켰다[14]. Virtual simulation 소프트웨어(vSim for Nursing, Laerdal Medical & Wolters Kluwer)를 활용한 내•외과 가상현실 시뮬레이션 교육은 간호 대학생의 간호과정 수행능력을 향상시켰다[15]. vSim for Nursing 소프트웨어의 소아간호 모듈을 사용한 가상현실 시뮬레이션 교육은 비판적 사고성향, 임상수행능력, 실습만족도를 향상시켰다[16]. 또한, 가상현실과 모바일 기기를 활용하여 시나리오 기반 입원관리 가상현실(scenario-based admission management virtual reality을 활용한 실습교육은 학습몰입감, 학습자신감, 학습만족도를 높인 것으로 나타났다[17]. 가상현실 간호교육 효과를 확인한 12개 연구를 메타 분석한 결과[18], 가상현실 간호교육은 지식 향상에 효과를 보였으나 기술, 만족도, 자신감, 수행시간에는 효과가 없었다[18]. 그리고 가상현실 기반 임상간호교육은 지식과 학습만족도를 높였으나, 자기효능감에는 효과를 보이지 않았다[19].

가상현실을 이용한 실습교육은 학습만족도, 자신감, 몰입감, 성취도의 장점으로 인하여 최근 5년간 간호 대학생의 지식, 기술, 태도 등의 다양한 주제로 보고되고 있다[20]. 모아간호 분야에서도 가상현실을 이용한 교육 중재가 시도되고 있으므로, 교육의 효과에 대하여 평가하고 추후의 연구 방향을 제시할 필요가 있다. 그러므로 본 연구에서는 가상현실을 이용한 모아간호 실습교육이 간호 대학생의 실습역량에 미치는 영향의 분석 연구들을 체계적으로 고찰하고자 한다. 체계적 문헌고찰은 모아간호 가상현실 교육 중재의 다양한 주제를 탐색하고, 최적의 기술적 방법론에 대하여 파악하며, 간호 대학생 실습역량에 미치는 영향을 확인할 수 있게 해 준다. 이는 대면 교육의 한계를 극복하여 가상현실 교육 중재를 계획하고 수행하는 간호 실무에 실제적 시사점을 제공할 수 있는 유용한 자료가 될 것이다.

본 연구의 목적은 가상현실을 이용한 모아간호 실습교육이 간호 대학생의 실습역량에 미치는 영향을 조사한 분석 연구(analytic studies)를 체계적으로 고찰하여 교육의 특성과 내용을 분석하고 유효성을 평가하는 것이다. 구체적인 목표는 다음과 같다. 첫째, 가상현실을 이용한 모아간호 실습교육의 방법을 파악한다. 둘째, 가상현실을 이용한 모아간호 실습교육의 주제를 파악한다. 셋째, 가상현실을 이용한 모아간호 실습교육이 간호학생의 실습역량에 미치는 효과를 평가한다. 넷째, 가상현실을 이용한 모아간호 실습교육이 간호학생의 심리•사회특성에 미치는 효과를 평가한다.

## Methods

Ethics statement: This study was exempted by the Institutional Review Board of Kongju National University as this study analyzed existing literature.

### 연구 설계

본 연구는 가상현실을 이용한 모아간호 실습교육 중재 연구의 일반적 특성, 교육 주제, 교육 방법, 간호 대학생의 실습역량에 미치는 영향을 평가한 체계적 문헌고찰 연구이다. 고찰 과정은 2020년 개정된 PRISMA (Preferred Reporting Items of Systemic Reviews and Meta-Analysis) 2020 statement [21]와 2022년 개정된 Cochrane Handbook for Systematic Reviews of Interventions version 6.3 [22]의 체계적 문헌고찰 보고지침에 따랐다.

### 문헌 검색

문헌 검색은 2명의 연구자가(Hwang과 Kim) 독립적으로 검색하였다. 문헌은 Cochrane Library, CINAHL, EMBASE, ERIC, PubMed, Research Information Sharing System (RISS)의 6개 검색 엔진으로부터 검색하였다. 검색 시기는 2022년 2월 4일부터 2월 15일 사이에 전자 자료원에서 검색어로 MeSH와 EMTREE와 virtual simulation과 같은 자연어, augmented reality와 같은 유사어, VR과 같은 약어를 이용하여 검색하였다. 문헌 검색은 Participant Intervention Comparison Outcome Setting Time-Study Design (PICOST-SD)의 평가질문을 기초로 고급 검색을 시행하였다[23]. 각 연구자가 모든 과정에서 사례보고서를 작성하여 독립적으로 문헌을 추출하고 평가하였고, 최종 자료는 연구자 회의를 통하여 일치도를 확인하였다. 검색원의 선정 기준은 National Library of Medicine의 COSI (COre Standard, Ideal)에서 제시한 Cochrane, PubMed, EMBASE의 핵심 데이터베이스와 CINAHL, ERIC, RISS, 수기 검색의 표준 영역으로 하였다[24]. 본 연구의 핵심질문은 “가상현실을 이용한 모아간호 실습교육은 간호학생의 실습역량을 향상시키는가?”와 “가상현실을 이용한 모아간호 실습교육은 간호학생의 심리•사회특성에 긍정적 영향을 주는가?”이다. 선정기준은 1) 간호 대학생인 전문학사, 학사 학생을 대상으로 한 연구, 2) 가상현실 중재, 3) 유사 실험연구, 무작위 실험연구, 단일군 전후설계 연구, 4) 영어나 한국어로 된 저널 논문, 5) 동료 평가로 진행된 학술 저널의 전문 이용 가능 논문, 6) 2022년 검색 시점까지 출판된 논문이었다. 제외 기준은 1) 대상자가 간호 대학생이 아닌 연구, 2) 연구 결과가 제시되지 않은 논문, 프로토콜 연구, 3) 회색 논문(학술대회 발표 논문, 연구 보고서), 학위논문, 단행본, 출판 진행 논문이었다. 검색 전략으로 국외 검색 데이터베이스는 Cochrane Library, CINAHL complete에서 “((“Students, Nursing”[Mesh] OR “Nursing”[Mesh] OR “Education, Nursing”[Mesh]) OR (Nursing Student[Title/Abstract] OR Nursing Students[Title/Abstract] OR Nursing[Title/Abstract])) AND ((“Virtual Reality “[Mesh]) OR (Augmented Reality[Title/Abstract] OR Mixed Reality[Title/Abstract] OR VR[Title/Abstract] OR AR[Title/Abstract] OR MR[Title/Abstract] OR Virtual Simulation[Title/Abstract] OR Gam*[Title/Abstract] OR Game Based Learning[Title/Abstract] OR Gamification[Mesh] OR Second World[Title/Abstract] OR Mirror World[Title/Abstract] OR 3D Environ*[Title/Abstract] OR videogam*[Title/Abstract]) AND (“Maternal-Child Nursing”[Mesh] OR Maternity Nursing[Title/Abstract])”로 하였고, 연구의 비뚤림 감소를 위하여 수기 검색을 시행하였다. 국내 문헌 검색은 RISS에서 ‘가상현실 간호, 메타버스 간호, 혼합현실 간호, 증강현실 간호’ 등의 키워드를 적용하였다. 문헌의 선정은 다음의 PICOST-SD의 핵심 질문에 따랐다.

#### 대상

연구의 대상자는 간호 대학생으로 하였으며, 간호사를 대상으로 한 연구는 제외하였다.

#### 중재

중재는 가상현실을 이용한 모아간호 실습교육으로 하였으며, 증강현실, 혼합현실, second life, mirror world, 3-dimensional environment, meta-verse를 이용한 교육을 포함하였다. 시뮬레이터만을 활용한 연구는 배제하였다.

#### 비교 대상

비교 대상은 대조군에게 가상현실 교육을 적용하지 않고 대면 교육을 제공한 연구였다. 비교 대상이 없이 가상현실 교육의 효과를 검증하는 단일군 연구도 선정하였다.

#### 결과

연구 결과로 간호 대학생의 실습역량으로 설정하였고, 실습역량은 지식, 기술, 태도를 포함하였다[12]. 일차변수는 지식, 기술, 태도의 직접적 간호역량으로[12], 이차변수는 교육으로 인한 심리•사회특성으로 불안, 자신감, 만족감, 자아효능감 등이 포함되었다.

#### 장소

장소는 가상현실 비대면 원격 교육이었고, 다양한 교육을 혼합한 경우, 주요 교육이 가상현실로 이루어진 연구는 포함하였다.

#### 평가 시점

교육 후에만 결과를 측정하거나, 교육 전후의 결과 측정 연구를 모두 포함하였다. 또한, 결과를 반복 측정한 연구도 포함하였다.

#### 연구 설계

연구 설계는 가상현실 교육을 적용한 분석 연구를 포함하였다. 즉 실험 연구, 코호트 연구, 환자대조군 연구였으며, 무작위 실험연구, 통제 전후 연구(controlled before and after study), 단속 시계열 연구(interrupted time series), 유사 실험연구(quasi experimental study)가 포함되었다[23]. 프로토콜 연구는 제외하였다.

### 문헌의 질 평가

선택된 문헌의 최종 질 평가는 2016년 발표된 Risk-Of-Bias In Non-randomized Studies of Interventions (ROBINS-I) [25]와 Risk of Bias 2 (RoB 2) [26] 도구를 활용하여 시각화하였다[27]. ROBINS-I은 Cochrane 연합에서 비무작위 연구의 비뚤림 평가를 위하여 개발한 질 평가 도구로서, 코호트 연구, 환자-대조군 연구, 통제 전후 연구, 단속 시계열 연구, 완전한 무작위 배정에 미치지 못하는 유사 실험연구, 관찰 연구를 평가할 수 있는 도구이다. ROBINS-I은 8개의 정해진 비뚤림 영역에서 판단에 도움이 되는 신호질문(signaling questions)에 따라 비뚤림 위험을 평가하는 방법을 제공해 준다. 8개의 평가 영역은 교란으로 인한 비뚤림 평가, 연구 대상자 선택의 비뚤림, 중재 분류의 비뚤림, 의도한 중재에서 이탈되어 나타나는 비뚤림, 결측치로 인한 비뚤림, 중재 결과 측정 비뚤림, 보고된 연구 결과 선택의 비뚤림, 전체 비뚤림 위험 판단이다. 각 영역은 2명의 연구자들이 독립적으로 코딩지와 질 평가 도구 매뉴얼을 활용해[25] 평가하였다. 세부 신호질문에 대한 평가로서 비뚤림의 위험은 낮음, 중등도, 높음, 매우 높음, 정보 없음 중에 해당하는 결과를 문헌별로 기록하였다. 1영역의 8가지 신호질문은 중재 효과의 교란 가능성, 추적 관찰기간의 구분, 중재의 중단이나 교환의 의미, 교란을 통제하는 분석 방법, 교란영역의 통제, 중재 후 변수의 통제, 시간-변동 교란을 보정하는 분석, 교란 영역의 보정에 대한 질문이다. 2영역의 5가지 신호질문은 연구 대상자의 선택, 선택에 영향을 준 변수와의 관련성, 선택과 중재 결과의 영향, 추적 관찰과 중재의 시작 시점, 선택 비뚤림을 교정하는 보정 방법이다. 3영역의 신호질문은 중재군의 정의, 중재군을 정의하는 정보, 중재 결과가 중재 분류에 미치는 영향에 대한 질문이다. 4영역의 신호질문은 중재의 이탈, 이탈이 중재 결과에 미치는 영향, 공동 중재의 중재 군간 균형, 중재의 성공적 시행, 배정된 중재법의 준수, 중재 준수의 효과를 추정하는 분석에 대한 질문이다. 5영역의 신호질문은 모든 대상자의 결과 자료 사용, 중재 상태에 대한 결측 자료, 결측 자료로 인한 대상자의 배제, 결측 연구자의 비율이나 원인, 결측치에 관계없는 연구 결과의 견고성에 대한 질문이다. 6영역의 신호질문은 결과 측정이 중재의 지식에 의해 받은 영향, 평가자들의 중재에 대한 인식, 중재 결과 평가 방법의 유사성, 중재 결과 측정의 체계적 오류에 대한 내용이다. 7영역의 신호질문은 중재 결과 영역 내에서 여러 개의 중재 결과 측정, 중재-결과 관계에서의 다중 분석, 다른 하위군의 중재 결과 보고에 대한 내용이다. 8영역은 1–7영역의 결과를 통하여 연구의 전체적 비뚤림을 평가하는 것이다.

RoB 2는 Cochrane 연합에서 무작위 실험연구의 질 평가를 위하여 개발한 도구로, 무작위 배정 과정에서 발생하는 비뚤림, 의도한 중재에서 이탈로 인한 비뚤림, 중재 결과 자료의 결측으로 인한 비뚤림, 중재결과 측정의 비뚤림, 보고된 연구 결과 선택의 비뚤림 등 전체 평가의 6가지 영역으로 되어 있다. 연구자는 각각 독립적으로 무작위 실험연구를 평가하고 비뚤림의 위험이 낮음, 높음, 불확실 중에 해당하는 결과를 기록하였다[26]. 평가 결과에 대한 연구자간 일치도는 kappa 계수를 사용하여 0.80 이상의 강한 일치도를 보이는 영역만 채택하였다[23].

### 자료 분석

선정된 최종 7편의 논문은 각 논문의 분석틀을 엑셀 프로그램을 사용하여 사례보고서로 작성하여 정성적 분석을 시행하였다. 사례 보고의 항목은 일반적 특성(제1저자, 연도, 국가, 중재 장소, 연구 설계, 대상자 특성, 대상자의 최종 분석 수, 대상자 선정기준), 교육 방법(교육 명칭, 형태, 대조군 교육, 매체, 기간, 회수), 교육 주제(주제, 1차 결과변수, 2차 결과변수, 측정 도구), 교육 효과의 유효성(실수, 백분율, 평균, 표준편차, *p*값)이었다.

## Results

### 가상현실을 이용한 모아간호 실습교육의 일반적 특성

**Figure 1.**
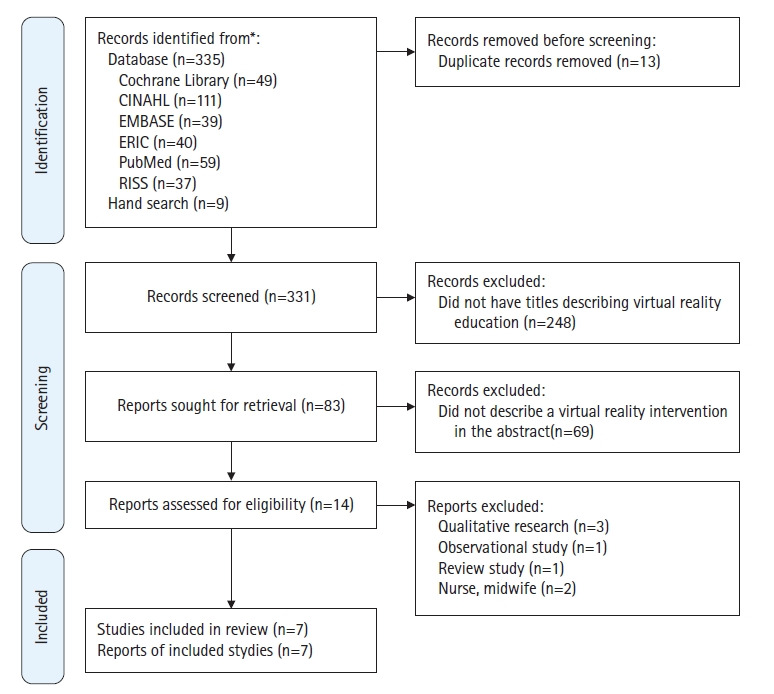
PRISMA 2020 flow diagram for the literature search.

검색된 논문의 수는 335편(Cochrane Library, 49; CINAHL, 111; EMBASE, 39; ERIC, 40; PubMed, 59; RISS, 37)이었으며, 검색된 논문들은 1984년부터 2021년 사이에 발행되었다. 참고문헌 목록과 Google Scholar로부터 수기 검색으로 9편의 논문을 추가하여 344편이 검색되었다. 이들의 연구 제목, 저자, 출판연도의 선택조건으로 한 문헌 목록으로부터 중복문헌 13편을 제거하여 331편이 남았다. 이들의 제목을 모두 읽어 가상현실이 아닌 248편을 제거하여, 83편이 남았다. 이들의 초록을 읽어 적합하지 않은 것으로 밝혀진 69편이 제거되어 14편이 남았다. 14편 문헌의 전문(full text)을 검토한 결과 질적연구 3편[28-30], 조사연구 1편[31], 고찰연구 1편[32], 대상자가 가족 전문 간호사학생인 1편[33], 조산사 과정 학생인 1편[34] 등 총 7편을 제거하여 최종 7편[35-41]을 추출하였다(Figure 1). 선정된 논문의 시기는 2014년 1편(14.3%) [35], 2016년 2편(28.6%) [36,37], 2020년이 1편(14.3%) [38], 2021년이 3편(42.9%) [39-41]이었다. 국가별로는 미국[35,40], 캐나다[37,39], 한국[38,41]이 각각 2편씩 있었고, 인도가 1편(14.3%) [36]이었다.

대상자의 최종 표본 수는 최소 35명에서[39] 최대 111명이었다[40]. 대상자의 학년은 2학년 2편(28.6%) [35,39], 3학년 2편(28.6%) [37,40], 4학년 2편(28.6%) [36,41], 명시하지 않은 연구 1편(14.3%) [38]이었다. 연구 설계는 단일군 사전사후 실험연구가 3편(42.9%) [35,36,38], 유사 실험연구가 2편(28.6%) [39,41], 무작위 실험 연구가 1편(14.3%) [37], 코호트 연구가 1편(14.3%) [40]이었다. 중재 장소는 가상 시뮬레이션 센터가 4편(57.1%) [37-39,41], 가상 시뮬레이션 클래스룸이 2편(28.6%) [35,36], 학습관리 시스템(learning management system, LMS)이 1편(14.3%) [40]이었다 (Table 1).

### 가상현실을 이용한 모아간호 실습교육 방법

중재 명칭은 ‘VPM (virtual pregnancy model)’ [35], ‘HirNIC VR (high-risk neonatal infection control)’ [41], ‘virtual LMS’ [40], ‘MNH (maternal and newborn health)’ [36], ‘newborn virtual simulation’ [39], ‘VCS (virtual clinical simulation)’ [37], ‘vSim for Nursing (virtual simulation for nursing)’ [38]의 7편이었다. 교육 형태는 원격 웹 기반 중재가 7편이었다. 대조군 교육은 대면 임상 실습이 2편, 전통적 교내 실습이 1편 [39], 대면 시뮬레이션 실습이 1편[37], 대면 임상실습이 2편[40,41]이었고, 대조군이 없는 연구가 3편[35,36,38]이었다. 매체는 가상현실 시뮬레이션 프로그램이 4편[37-39,41], 유튜브 가상현실 프로그램이 1편[35], 가상현실 LMS가 1편[40], virtual classroom objective structured clinical examination (OSCE)가 1편[36]이었다. 중재기간은 최소 72시간에서[36] 최대 1학기[37,39]였으며, 중재회수는 최소 1회에서[35] 최대 5회[38]였다(Table 2).

### 문헌의 질 평가 결과

**Figure 2.**
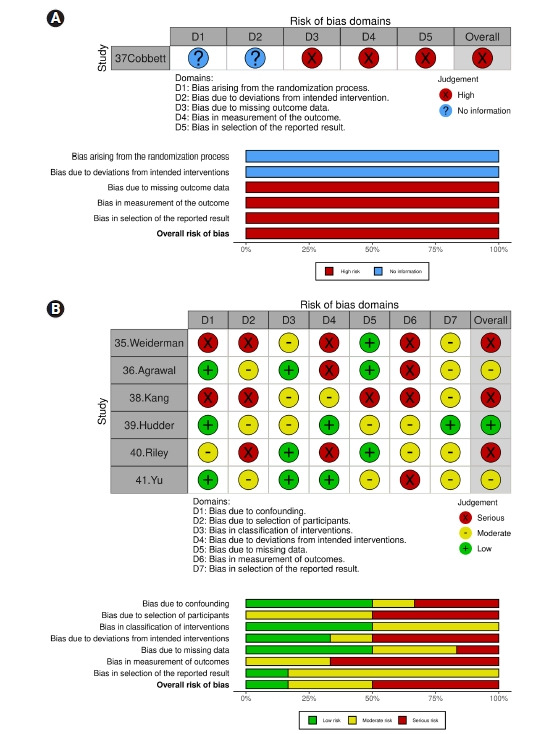
Risk of bias graph and summary for randomized controlled trials (RCTs) (A) and non-RCTs (B).

불일치가 나타나는 모든 항목은 연구자 회의를 거쳐 논문을 재검토하여 질 평가를 확정하였다. 1편의 무작위 연구는[37] RoB 2로 평가하였고, 나머지 6편[35,36,38-41]의 ROBINS-I 평가 결과는 질 평가 시각화 도구인 Risk-of-bias VISualization (robvis)의 신호등 차트와 막대 도표로 제시하였다[27] (Figure 2). RoB 2를 사용한 무작위 연구 1편의 전체 평가 결과 1, 2영역에서 정보가 없고 3–6영역에서 비뚤림 위험이 높았다. ROBINS-I를 사용한 비무작위 연구 6편의 질 평가 종합 결과 3편이 높음, 2편이 중등도, 1편이 낮음이었다. 교란으로 인한 비뚤림 평가는 2편이 높음, 1편이 중등도, 3편이 낮음이었다. 연구 대상자 선택의 비뚤림은 3편이 높음, 3편이 중등도였다. 중재 분류의 비뚤림은 3편이 중등도, 3편이 낮음이었다. 의도한 중재에서 이탈되어 나타나는 비뚤림은 3편이 높음, 1편이 중등도, 2편이 낮음이었다. 결측치로 인한 비뚤림은 1편이 높음, 2편이 중등도, 3편이 낮음이었다. 중재 결과 측정 비뚤림은 4편이 높음, 2편이 중등도였다. 보고된 연구 결과 선택의 비뚤림은 5편이 중등도, 1편이 낮음이었다(Figure 2). 두 명 연구자 간 평가 합치도는 kappa 계수 0.80–1.00이었다.

### 가상현실을 이용한 모아간호 실습교육 주제

교육 주제는 신생아 소생술과 간호가 1편[36], 신생아 일반 간호와 피부간호가 1편[41], 임신과 신생아 간호가 1편[35], 임신성 당뇨 여성의 출산간호와 신생아 간호가 1편[40], 신생아 호흡간호, 위생술, 환경관리가 1편[39], 자간전증 임신간호가 1편[37], 그리고 정상 임부의 간호가 1편[38]이었다(Table 2).

교육의 1차 결과변수는 신생아 간호 지식이 2편[39,41], 자간전증과 감염 간호지식이 1편[37], 학습능력(여성의 요구 예측능력, 임신 불편감 인식, 위험신호 인식, 차이점 인식)이 1편[35], 고위험신생아 간호수행도가 1편[40], 모아간호 수행도가 1편[36]이었다. 2차 결과변수는 만족도가 2편[39,40], 자신감이 2편[37,39], 교육요구도[36], 불안[37], 비판적 사고[38], 자아효능감[41], 자기주도 학습이 각각 1편[38]이었다(Table 3). 결과변수의 측정도구는 시험을 통한 지식 점수[37], 만족도[40,41], 자아효능감[41], 기술 체크리스트 점수[36], 간호수행도[40] 등이었다(Table 2).

### 가상현실을 이용한 모아간호 실습교육이 지식, 기술, 태도에 미치는 영향 평가: 1차 결과변수

가상현실을 이용한 모아간호 실습교육은 간호지식 점수에 부분적으로 긍정적 효과를 나타냈다. 신생아 감염관리 지식은 실험군 23.44(±2.15)점, 대조군 23.29(±1.92)점으로 통계적으로 유의한 차이를 나타내지 않았다(*p*=.213) [41]. 자간전증 간호지식은 실험군 4.12(±1.54)점, 대조군 4.80(±1.19)점으로 통계적으로 유의한 차이가 없었고(*p*=.09), 감염지식도 실험군 6.40(±1.73)점, 대조군 6.82(±1.25)점으로 통계적으로 유의한 차이가 없었다(*p*=.31) [37]. 신생아 간호사정 지식은 실험군 2.80(±1.90)점, 대조군 1.18(±2.09)점으로 실험군이 통계적으로 유의하게 높았다(*p*=.03) [39]. 임신 간호지식은 사전 점수 91.10(±22.03)점, 사후 점수 183.68(±12.87)점으로 중재 후 통계적으로 유의하게 증가하였다(*p*<.01) [35] (Table 3).

가상현실을 이용한 모아간호 실습교육은 간호기술 점수에 부분적으로 긍정적 효과를 나타냈다. 신생아 간호기술 OSCE 평균이 사전 21.3점에서 사후 62점으로 통계적으로 유의한 차이가 있었다(*p*=.001) [36]. 출산 간호기술은 실험군 51.46(±3.52)점, 대조군 51.44(±3.77)점으로 통계적으로 유의한 차이가 없었다(*p*=.95) [40] (Table 3).

### 가상현실을 이용한 모아간호 실습교육이 심리•사회특성에 미치는 영향 평가: 2차 결과변수

가상현실을 이용한 모아간호 실습교육은 실습만족도 점수에 부분적으로 효과를 나타냈다. 교육만족도는 실험군 4.79(±0.35)점, 대조군 4.13(±0.47)점으로 실험군이 통계적으로 유의하게 높았다(*p*<.001) [41]. 만족도가 실험군 4.53점, 대조군 4.70점으로 통계적으로 유의한 차이가 없는 결과도 있었다(*p*=.24) [40] (Table 3).

가상현실을 이용한 모아간호 실습교육은 자신감 점수에 긍정적 효과를 나타내지 않았으나, 자아효능감에 긍정적 효과를 나타냈다. 자신감이 실험군 49.08(±5.42)점, 대조군 56.23(±6.35)점으로 대조군이 통계적으로 유의하게 높았다(*p*<.001) [39]. 자신감이 실험군 104.89(±17.52)점, 대조군 115.25(±21.95)점으로 통계적으로 유의한 차이가 없는 결과도 있었다(*p*=.059) [37]. 자아효능감은 실험군 8.57(±0.98)점, 대조군 7.72(±1.37)점으로 실험군이 통계적으로 유의하게 높았다(*p*=.018) [41] (Table 3).

가상현실을 이용한 모아간호 실습교육은 교육요구도에 긍정적 효과를 나타냈고, 불안에 부정적 효과를 나타냈으며, 비판적 사고, 자기주도 학습에는 효과를 나타내지 않았다. 교육요구도는 실험군의 100%, 대조군의 22.8%에서 만족하여 통계적으로 유의한 차이가 있었다(*p*<.001) [36]. 불안은 실험군 73.26(±19.95)점, 대조군 57.75(±15.25)점으로 실험군이 통계적으로 유의하게 높았다(*p*=.001) [37]. 비판적 사고는 사전 98.83(±9.44)점, 사후 97.96(±9.81)점으로 통계적으로 유의한 차이가 없었다(*p*=.872) [37]. 자기주도 학습은 사전 154.91점(±17.89)점, 사후 155.45(±16.44)점으로 통계적으로 유의한 차이가 없었다(*p*=.881) [38] (Table 3).

## Discussion

문헌의 체계적 문헌고찰 결과 선정된 7편 논문이 2014–2021년에 발표되었고, 이 중 4편이 최근 2년 사이(2020–2021) 발표되어 가상현실을 이용한 교육이 최근 활발하게 이루어지고 있음을 알 수 있었으며, 온라인 기반 교육의 증가 추세와 함께 간호학 실습교육 요구도가 향후에도 증가될 것으로 예측된다는 보고를 반영하였다[20]. 본 장에서는 가상현실을 이용한 모아간호 실습교육이 어떠한 방향으로 나아가야 하는지 함의를 파악하기 위해 연구의 방법, 질, 주제, 유효성에 관하여 논하고자 한다.

고찰된 연구의 방법으로는 유사 실험연구가 많았고, 무작위 실험연구도 비뚤림이 높았기에 연구 방법론적인 질이 낮았다. 그러므로 근거 수준을 높이기 위한 무작위 배정 맹검 실험연구가 축적될 필요가 있다. 대상자는 간호 대학생 2–4학년이 각각 2편씩으로 고르게 분포되어 교육과정의 넓은 범위에서 사용할 수 있다는 점을 시사한다. 중재의 기간에서 자유롭게 접속 가능한 중재는 없었다. 의학, 간호학 학생의 이러닝 사용 연구에 따르면 90.7%의 학생이 이러닝 강의내용을 자유롭게 공유하여 학습에 도움을 받고자 하는 것으로 나타났으므로[39], 가상현실 프로그램에 언제 어디서나 접속하여 실습역량을 향상할 수 있도록 하는 것이 필요하다[42].

본 연구에서 고찰된 문헌의 질 평가 결과, 교육 효과의 교란변수를 통제하는 위험이 있었으므로 추후 연구에서는 중재 효과의 비뚤림을 상쇄하기 위해 분석과정에서 회귀분석, 층화분석, 짝짓기, 가중치 적용 등의 통계적 방법이 필요할 것이다[23]. 연구 대상자의 선택 비뚤림은 6편에서 높거나 중등도로 나타났다. 그러므로 추후 연구에서는 대상자 선택에서 교육 시작 전에 관찰된 특성에 대하여 동질성 검정, 공변량 분석 등이 필요할 것이다[23]. 또한 중재 분류의 비뚤림 평가에서는 중등도가 4편에서 나타나, 교육 시작 전에 프로토콜 단계에서 중재의 유형, 장소, 빈도, 강도, 시기가 명확하고 명시적이어야 할 것이다. 의도한 교육에서 이탈되어 나타나는 비뚤림에 대한 질 평가는 3편에서 높음으로 나타났다. 그러므로 교육자는 의도한 대로 중재가 시행되도록 점검하고, 중재군 간의 탈락률이 불균형하거나, 탈락률이 높지 않도록 관리해야 할 것이다. 결측치로 인한 비뚤림은 높거나 중등도인 연구가 3편으로 상대적으로 양호하였으나 향후 연구에서는 95% 이상의 참여자가 교육에서 이탈되지 않도록 관리하고, 완료되지 않은 경우의 사례에 대한 분석이 필요하다. 마지막 영역인 중재 결과 비뚤림의 평가에서는 낮음으로 평가된 연구가 없었으므로, 향후 연구는 이중맹검 무작위 연구가 바람직한 연구방향이 될 것이다. 보고된 연구 결과 선택의 비뚤림 영역에서는 중등도가 5편으로 나와 결과의 다수 효과 추정치를 보고하고 프로토콜 연구가 선행될 필요[25]를 입증한다. 전체 질 평가 결과 비뚤림 위험이 높은 편이므로 향후 연구에서는 방법론적인 세부 사항을 엄격하고 완전하게 보고해야 할 것이다.

중재의 주제는 대부분 신생아를 대상으로 하고 있어[35,36,39,40], 다양한 대상의 모아간호 주제가 개발될 필요가 있다. 대부분의 교육이 신생아에 집중 또는 포함함에 비해 임신 및 출산 여성에 초점을 맞춘 교육은 3편으로 적었고[37,38,40], 특정 질환으로는 자간전증, 임신 중 감염, 임신성 당뇨의 주제만이 발견되었다. 다양한 모아간호 분야가 가상현실 중재로 적용되지 않고 있어, 추후에 조기진통, 난산과 같은 고위험 임신 관련 가상현실 간호교육이 개발될 필요가 있다[20]는 지적을 지지한다. 중재의 효과는 지식과 기술에 효과를 나타낸 연구들이 일부 존재하였으므로[35,36,39] 실습역량 향상에 도움을 줄 것으로 예측된다. 본 연구의 지식 결과 변수에서는 신생아 간호지식[39]과 임신 간호지식[35]에서만 효과를 보였으나 이는 교육의 반복 제공 여부와 관련된 것으로 유추된다. 유튜브를 통한 제공[35], 한 학기 동안의 제공[38]이 다른 연구에 비해 교육 제공의 반복이 가능하고, 중재 노출 기간이 길었기 때문으로 보인다. 타 간호 학문 분야에서 가상현실을 이용한 간호교육의 효과를 메타 분석한 연구에서는 지식 향상에 효과가 있었음을 보고하였므로[18], 모아간호 분야에서도 지식에 유효할 가능성이 있으므로 관련 연구가 축적되어야 한다.

심리•사회적 효과면에서는 학생들의 교육요구도[38], 만족도[41], 자아효능감[41]이 높았으므로, 실습교육에 긍정적인 효과를 준다는 것이 확인되었다. 하지만 대면 실습에 비해 가상현실을 이용한 교육에서 불안감이 높다는 연구[37]를 고려하면, 간호 대학생이 심리적인 어려움을 경험할 수 있음을 알 수 있다. 본 연구에도 자신감[38]이 낮았던 결과가 보였는데, 이는 선행연구에서 메타 분석한 결과[18]와도 같은 맥락이다. Chen 등[18]은 모든 간호분야의 가상현실 적용 교육 효과를 12편에서 메타 분석하였는데, 지식에만 효과가 있고 기술, 수행시간, 자신감, 만족도에도 차이가 없었다고 보고하였다. 가상현실에 학생들이 빠르게 적응하는 것은 부담이 되므로, 전통적 교육을 혼합하여 제공하는 것이 좋다[2]. 또한, 가상현실은 역동적이고 도전적이므로 몰입하기 이전 단계에서 접근의 심리적 장애를 제거하는 ice breaking 등이 필요하다[18]. 그러므로 가상현실을 이용한 교육을 계획할 때 불안을 감소시킬 수 있도록, 사전 교육, 안내와 교수자의 심리적인 지지가 중요함을 알 수 있다.

이와 같이 가상현실을 이용한 교육의 증가에도 불구하고 모든 실습역량에 효과가 있었던 것이 아니므로, 임상현장 교육을 기본으로 제공하고, 가상현실에서 효과가 있었던, 지식, 기술, 만족도, 자아효능감의 향상을 목표로 설정하는 것이 바람직할 것이다. 가상현실을 이용한 모아간호 실습교육의 방법을 살펴보면, 전통적인 LMS 기반의 온라인 교수학습센터를 활용한 연구도 1편 있었지만[40] 주로 가상현실 프로그램 재현을 위한 센터와 웹사이트가 구축되어 있음을 알 수 있다[35-39,41]. 그러므로 가상현실 교육을 위한 웹이나 플랫폼 구축이 선행되어야 하므로 교육 방법의 인터페이스 구축이 기본 요건임을[2] 알고 전략을 세워야 할 것이다.

본 연구의 의의는 첫째, 가상현실을 이용한 모아간호 실습교육의 경향과 유효성을 확인할 수 있었다는 점이다. 둘째, 간호교육자가 가상현실 실습 교육을 시행하였을 때, 간호 대학생의 지식, 기술, 만족도, 교육요구도, 자아효능감 증진에 유효하였다는 근거를 확인할 수 있다는 점이다. 셋째, 모아간호 분야에서는 주로 신생아 간호실무의 주제에 대하여 연구되어 정상임신, 분만 및 고위험 모아간호 분야의 가상현실을 이용한 교육을 개발할 필요성을 확인하였다는 점이다. 넷째, 가상현실을 이용한 실습교육이 간호 대학생에게 불안을 높이고 자신감을 저하시킬 수도 있으므로 체계적인 교육 프로그램 설계가 필요하다는 점이다.

본 연구의 한계점으로는 첫째, 고찰된 문헌의 언어가 영어로만 되어 있는 점, 둘째, 학위논문 등의 회색 문헌을 포함하지 않았으므로 출판 비뚤림이 있다는 점이다.

본 연구는 가상현실을 이용한 모아간호 실습교육이 간호 대학생 실습역량에 미치는 영향의 실험연구를 체계적으로 고찰하는 연구로서, 7편의 문헌을 분석하였다. 교육의 특성과 내용을 분석하고 유효성을 평가한 결과, 교육은 자간전증, 감염 임부, 당뇨 임부, 출산 산부, 신생아를 대상으로 가상현실 플랫폼이나 웹사이트를 통하여 제공되었음을 알 수 있다. 고찰 결과 간호 대학생의 지식, 기술, 참여도, 만족도, 교육요구도, 자아효능감에도 긍정적 효과를 보였다. 하지만 간호 대학생의 불안, 자신감에는 부정적인 효과를 보였다. 문헌들은 전체 비뚤림 위험이 높은 연구가 4편이었고, 세부 영역의 비뚤림 위험은 낮음, 중등도, 높음이 고르게 분포하였으나, 문헌의 수가 많지 않아 일반화에는 어려움이 있다. 본 연구를 통하여 다음을 제언하고자 한다. 첫째, 문헌고찰 결과 가상현실을 이용한 모아간호 실습교육이 부족하였으므로, 임신, 분만, 고위험 모아간호 주제로 실습교육을 적용할 것을 제언한다. 둘째, 부분적으로 유효했던 결과에 대하여 방법론적으로 잘 설계된 실험연구를 통하여 검증할 것을 제언한다. 셋째, 본 연구의 결과를 토대로 간호 대학생에게 가상현실 실습교육을 위한 지침을 제공하여 지식, 기술, 태도에 긍정적 영향을 줄 수 있도록 세심하게 계획할 것을 제언한다.

## Figures and Tables

**Table 1.** Characteristics of selected studies (N=7)

First author (year)	Reference No	Country	Setting	Study design	Participants	No. of participants (Exp/Cont)
Weideman (2014)	[35]	United States	Virtual community	Single-group pre- and post-test	Second-year students	91
Agrawal (2016)	[36]	India	Virtual classroom	One-group pre- and post-test intervention design	Fourth-year students	83
Cobbett (2016)	[37]	Canada	Virtual simulation	Randomized controlled trials	Third-year students	55 (27/28)
Kang (2020)	[38]	Korea	Virtual simulation	One-group pre- and post-test study	Unspecified students	47
Hudder (2021)	[39]	Canada	Virtual simulation	Quasi-experimental design	Second-year students	35 (24/11)
Riley (2021)	[40]	United States	Learning management system	Two-group cohort study	Third-year students	111 (59/55)
Yu (2021)	[41]	Korea	Virtual simulation	Non-equivalent control group design	Third-year students	50 (25/25)

Cont: Control group; Exp: experimental group.

**Table 2.** Intervention characteristics of selected studies (N=7)

Author (year)	Reference No	Title of intervention	Intervention comparator	Intervention tool	Measurement scales	Time span	Number of sessions	Themes of intervention
Weideman (2014)	[35]	Virtual pregnancy model	-	YouTube virtual simulation	Four questions	Introductory clinical course	-	Introduction, live pregnancy virtual character and newborn
								
Agrawal (2016)	[36]	Maternal and newborn health	-	OSCE checklist	OSCE performance	72 hours	-	Labor management, newborn care, newborn resuscitation, partograph, infection prevention
Cobbett (2016)	[37]	Virtual clinical simulation	High-fidelity mannequin simulation (face to face)	vSim	Test & survey	Maternity nursing course	-	Caring for pregnant women with preeclampsia
Kang (2020)	[38]	vSim for Nurses	-	vSim	Survey	4-day class	5 phases	Maternal nursing scenarios
Hudder (202 1 )	[39]	Newborn virtual simulator	Traditional lab-based group	VR	Questionnaire & rubric	Fall semester	-	Respiratory assessment, evaluating environmental scans, hand hygiene
Riley (2021)	[40]	Virtual LMS	In person clinical learning	Combined simulation experience	Student performance	Course semester 7.5 weeks	Twice every spring semester	Infants of mothers with diabetes, women’s health, childbearing experience, maternal care, and neonatal care
					Student satisfaction			
Yu (2021)	[41]	High-risk neonatal infection control	Only NICU clinical practice	VR simulation program	HirNIC knowledge	90 minutes	5 sessions	Introduction, use of VR, basic care, feeding, skin care, and environmental management discussion
					Self-efficacy			
					Learner satisfaction			

HirNIC: high-risk neonatal infection control; LMS: learning management system; NICU: neonatal intensive care unit; OSCE: Objective structured clinical examination; VR: virtual reality.

**Table 3.** Outcomes of selected studies (N=7)

Author (year)	Reference No	Intervention format	Primary outcomes	Secondary outcomes	Experimental group,	Control group,	t or F	*p* or 95% CI
					Mean±SD or n (%)	Mean±SD or n (%)		
Weideman (2014)	[35]	Virtual patients	Learning competency	-	Total 91.10±22.03	Total 183.68±12.87	Total 34.67	Total <.01
			① Ability to anticipate women’s needs		①50.78±14.55	93.11±8.95	22.89	<.01
			② Discomfort of pregnancy		②24.95±10.26	42.09±7.38	12.16	<.01
			③ Warning signs		③31.54±9.99	48.69±3.70	16.30	<.01
			④ Differentiation		④24.29±12.84	66.15±5.92	29.60	<.01
Agrawal (2016)	[36]	Competency-based training using a virtual classroom	① Performance of maternal newborn nursing skill	② Educational needs improvement for VR	Pre	Post		①Pre <.001
					① 21.3	① 62		(95% CI, 19.9–22.6)
					② 83 (100.%)	② 21 (22.8%)		Post <.001
								(95% CI, 60.3–63.7)
Cobbett (2016)	[37]	Virtual simulation (vSim)	① Knowledge of preeclampsia	③ Anxiety	① 4.12±1.54	4.80±1.54	1.75	.09
			② Knowledge of group B *Streptococcus*	④ Self-confidence	② 6.40±1.73	6.82±1.25	1.02	.31
					③ 73.26±19.95	57.75±15.25	−3.2	.002
					④ 104.89±17.52	115.25±21.95	1.93	.059
Kang (2020)	[38]	Virtual simulation (vSim)	① Critical thinking	② Self-directed learning ability	① 98.83±9.44	97.96±9.81	0.439	.872
					② 154.91±17.89	155.45±16.44	–0.150	.881
Hudder (2021)	[39]	Virtual simulation film	① Knowledge of newborn assessment	② Satisfaction and self-confidence	① 2.80±1.90	1.18 ± 2.01	–2.27	.03
					② 49.08±5.42	56.23 ± 6.35	3.16	.001
Riley (2021)	[40]	Learning management system	① Performance of high-risk newborn care	② Satisfaction	① 51.46±3.52	51.44±3.77	t=.03	.95
					② 4.53 (-)	4.70 (-)	z=–2.26	(95% CI: 50.42–52.45)
								<.24 (r=.04)
Yu (2021)	[41]	Virtual reality simulation application	① Knowledge of newborn care	② Self-efficacy	① 23.44±2.15	23.29±1.92	272.00	.213
				③ Satisfaction	② 8.57±0.98	7.72±1.37	–2.16	.018
					③ 4.79±0.35	4.13±0.47	–5.59	<.001

VR: Virtual reality.
